# Effects of Maternal Exposure to Decamethylcyclopentasiloxane on the Alternations in Offspring Behaviors in Mice

**DOI:** 10.3390/biomedicines11010035

**Published:** 2022-12-23

**Authors:** Donglin Yi, Kangmin Kim, Minsu Lee, Eui-man Jung, Eui-Bae Jeung

**Affiliations:** 1 College of Veterinary Medicine, Chungbuk National University, Cheongju 28644, Republic of Korea; 2 Department of Molecular Biology, College of Natural Sciences, Pusan National University, Busan 46241, Republic of Korea

**Keywords:** decamethylcyclopentasiloxane, developmental neurotoxicity, behavior abnormality

## Abstract

D5, a member of the cyclic siloxane family, is widely used in personal care products such as shampoo, cosmetics, and deodorant and as an industrial intermediate. D5 can mainly be absorbed orally or through inhalation. Through these routes, people are exposed to D5 daily. However, the risk of prenatal exposure to D5 has not been fully elucidated. In this study, the effect of D5 on neural development was established through behavioral tests on offspring mice. The result confirmed that the maternal administration of 12 mg/kg of D5 showed depression in tail suspension and decreased performance in the forced swimming test as well as an increase in repetitive activity in both the marble-burying test and grooming test compared to the vehicle group. Furthermore, the 12 mg/kg group showed a decrease in cognitive ability and social behavior in the three-chamber test. In the novel object recognition test, memory impairment and a lack of exploring ability were found in the 12 mg/kg group. In conclusion, it is suggested that maternal D5 exposure has developmental neurotoxicity and can cause behavioral disorders in the offspring of mice. Thus, the usage of D5 needs to be considered carefully.

## 1. Introduction

Artificial chemicals are used widely to produce everyday products, such as detergent, medications, and food seasoning. The use of these chemicals has increased constantly since the 20th century. Decamethylcyclopentasiloxane (D5) is a siloxane family and is one of the most common chemical ingredients for daily necessities such as shampoo, lotion, sunscreen, and cosmetic products such as lipstick [1,2]. It was determined that 1,000,000~10,000,000 kg of D5 was imported into Canada, for instance, in complete products or as the pure chemical in 2006 [3]. As the usage of D5 is vast and varied, it is also disposed of into the environment [3]. D5 can be absorbed orally, and the systematical absorption of D5 may occur due to the inhalation of aerosol products. People are exposed to D5 daily [1,2]. The present study indicates that concerns about the safety of using artificial chemicals are well-founded because some of them have been revealed to be toxicants that can cause reproductive [4,5,6], metabolic [7], and developmental disorders [8]. They can disturb the endocrine [9,10], immune [11,12], and metabolic systems [7,12]. Recently, issues regarding endocrine-disrupting chemicals (EDCs) have been raised because humans could easily be exposed to them in our daily lives and very small quantities of EDCs can affect the function of organs. Current studies show that EDCs such as bisphenol A are suggested to cause and worsen autism spectrum disorder [13,14]. The correlation between EDCs and neurodevelopmental disorders such as autism spectrum disorder, schizophrenia, and attention deficit–hyperactivity disorder (ADHD) has been studied, and several chemicals with neurodevelopment toxicity have been prohibited owing to their serious side effects [7,14,15].

Octamethylcyclotetrasiloxane (D4), which has been identified as an EDC [15], has a similar structure to D5. The developmental neurotoxicity of D4 was reported in a previous study [16]. Due to the structural similarity of D4, the possibility of D5 developmental neurotoxicity needs to be evaluated. A previous study evaluated the toxicity of D5 in rats using an inhalation test. In the chronic exposure test, Fischer 344 rats (F344) showed a slight increase in liver weight in the early exposure period, but the liver adapted and quickly returned to its original weight [17]. A study evaluating exposure for consumers, workers, and the general public reported that around 0.6 mg/kg of D5 is systemically absorbed by humans per day [2]. Workers in specific occupations, such as hair designers, makeup artists, laundry workers, and dry-cleaning industry workers, are exposed to approximately 100 to 10,000 times more D5 than the average. Nevertheless, D5 has no estrogenic or androgenic effects in rats. In addition, the one-generation and two-generation tests showed that D5 did not affect the reproductive ability.

However, the effect of maternal D5 exposure on the neural development of their offspring is unknown. Therefore, this study examined the effect of maternal exposure to D5 on mouse offspring by studying the basal molecular mechanism.

## 2. Materials and Methods

### 2.1. Developmental Neurotoxicity Test (DNT)

To evaluate the effect of D5 on embryos, the developmental neurotoxicity of D5 was tested using the 46C mouse neural progenitor cell line (46C). Sox1 protein, which is a transcription factor and is known as a marker for the differentiation of neural progenitor cells, is tagged with GFP in the cell line. With DNT, the effect of D5 on the cell viability of undifferentiated neural progenitor cells can be evaluated. The effect of D5 on neural progenitor cell differentiation can also be evaluated by measuring the GFP fluorescence intensity of differentiated 46C cells in DNT. 46C was cultured in undifferentiated conditions in Dulbecco’s Modified Eagle’s Medium (Biowest, Rue de la Caille, Nuaille, France) with 15% fetal bovine serum (Biowest, Rue de la Caille, Nuaille, France) at 37 °C with 0.1% mouse leukemia inhibitory factor (Merk, Darmstadt, Germany). The medium was changed daily, and subculture was performed once every two days on a 100 mm culture plate. To evaluate the IC_50_ value of D5, the cell viability assay was performed in a 96-well clear-bottom plate. A total of 7000 cells were seeded in each well and 24 h after cell seeding, the media containing D5 (concentration range: 10^−9^ M~10^−2^ M) was treated with 200 μL in each well. Forty-eight hours after chemical treatment, a cell-viability assay was performed with media containing 10% CCK reagent (Dogenbio, Seoul, Republic of Korea). To evaluate the ID_50_ value of D5, the fluorescence intensity of GFP was detected in a 96-well, clear, round-bottom plate. In total, 100 cells were seeded in each well, and 4 days after cell seeding, 200 μL of differentiating condition media containing D5 (concentration range: 10^−9^ M~10^−2^ M) made with Dulbecco’s Eagle’s Medium F12 (GIBCO, Grand Island, NY, USA) and 15% fetal bovine serum (Biowest, Rue de la Caille, Nuaille, France) was treated in each well. Forty-eight hours after chemical treatment, the fluorescence intensity of GFP was detected with fluorescence spectroscopy (Lionheart FX). Data were analyzed with GraphPad Prism 8.

### 2.2. Animals

Twelve-week-old male and female C57BL/6N mice were purchased from Koatech (Pyeongtaek, Gyeonggi, Republic of Korea). Every surgical procedure and animal care process was approved by the Ethics Committee of the Chungbuk National University (Cheongju, Republic of Korea). The ethical approval code is CBNUR-1509-21. Mice were housed in a semi-specific pathogen-free area (semi-SPF) in the laboratory animal research center of Chungbuk National University (Cheongju, Republic of Korea). Mice were habituated for 1 week after arrival and exposed to light from 8 a.m. to 8 p.m. every day. Mice were mated from 6 to 9 p.m. Male and female mice were placed in a cage (200 × 260 × 130 mm, brown-colored polycarbonate) in a 1:3 ratio. After mating, the appearance of virginal plugs on female mice was considered a marker for pregnancy. Pups were weaned at 4 weeks after birth.

### 2.3. Chemical Treatments

**Figure 1:**
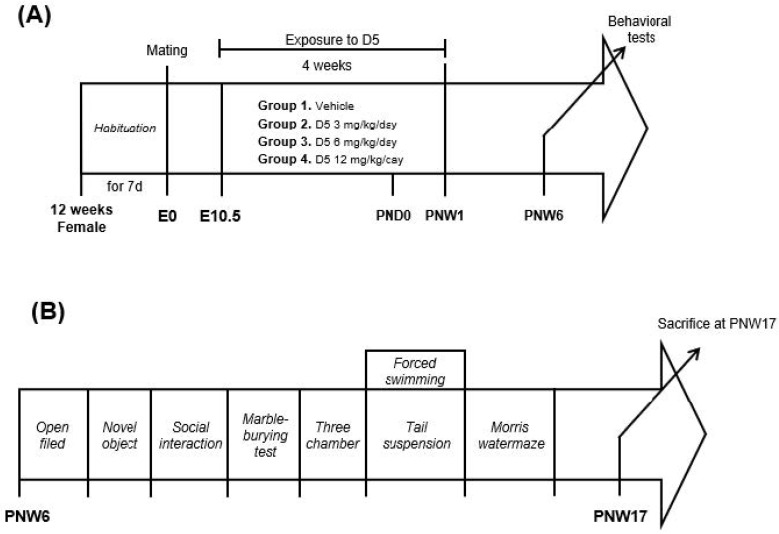
Experiment procedure: (**A**) maternal D5 exposure timetable; (**B**) behavioral test timetable.

D5 was treated with 200 mL corn oil vehicle as it is a lipophilic chemical and stored in adipose tissue [18]. The day on which a vaginal plug was observed in female mice was regarded as E0. D5 was treated from E10.5 through postnatal day 7 (Figure 1A). This study was designed to simulate real life. Thus, 6 mg/kg was set as a standard dosage (Health & Council, 2006). A measure of 3 mg/kg, half of the standard, was set as the low dose, and 12 mg/kg was set as the high dose. Each mouse was treated orally with D5 in corn oil with a 1 mL syringe fitted with a 20-gauge mouse oral needle.

### 2.4. Behavior Tests

Behavior tests were conducted when offspring mice were 6 weeks old (Figure 1B). Offspring mice were sacrificed 1 week later for the stabilization of the mouse conditions after their behavior was evaluated (Figure 1B).

#### 2.4.1. Grooming Test

To evaluate repetitive and compulsive behavior, the self-grooming time was measured. Each mouse was placed in a clear cage, and its behavior was recorded for 10 min. The accumulative time that each mouse spent grooming itself was measured.

#### 2.4.2. Marble-Burying Test

Twenty glass marbles (15 mm diameter) were laid on the surface of the 4 cm-deep paper bedding equidistant from each other in a 4 × 5 arrangement in each polycarbonate cage (25 × 40 cm width with 18 cm height). The mouse was placed into a corner of the cage and the experiment was carried out in a completely dark room. Mice were allowed to freely explore for 30 min. After the test, mice were removed from the cage and the number of buried marbles was counted. The marbles covered by at least 50% by bedding were considered to be buried marbles.

#### 2.4.3. Three-Chamber Test

The apparatus for the three-chamber sociability test consisted of three Plexiglass chambers with clear walls. Each chamber has a width of 40 cm, a length of 20 cm, and a height of 22 cm with small openings (10 × 5 cm) that allow mice to access each chamber. At first, for habituation, each experimental mouse was placed in the middle chamber and permitted to freely explore all three chambers for 5 min, which had two cylindrical empty cages (diameter of 8 cm with a height of 10 cm and grid wires) attached to both side chambers. For sociability testing, a stranger mouse (stranger 1, referred to as ‘S1’) was placed in a cylindrical plastic cage in one of the side chambers, and on the other side of the chamber, the cylindrical plastic cage remained empty (E). Then, the subject mouse was placed into the middle chamber and allowed to freely explore between three chambers for 10 min. For the social novelty test, the empty plastic cage was replaced with a wild-type stimulus mouse (stranger 2 (S2)) and the subject mouse was allowed to explore all three chambers for 10 min. The time spent around the cylindrical cages, the travelling distance, and heat maps were measured using the EthoVision XT 14 (Noldus, Wageningen, The Netherlands) for each experiment.

#### 2.4.4. Social Interaction Test

In the social interaction test, one stranger mouse and one subject mouse were placed in a chamber at the same time. The stranger mouse was the same species and same gender as the subject mouse but without chemical treatment. Both mice were free to explore the chamber and allowed to interact with each other. The behavior was recorded for 10 min. Following, sniffing, anogenital grooming, and fighting behavior was recorded throughout the experiment.

#### 2.4.5. Tail Suspension Test

Mice were suspended by their tails 50 cm above the ground. The experiment was recorded for 5 min, then the immobility time was measured.

#### 2.4.6. Forced Swimming Test

Each mouse was placed in a 2 L beaker filled with 1400 mL of water at 23~27 °C. Each experiment was recorded for 5 min, and the immobility time was measured with EthoVision XT 14 program.

#### 2.4.7. Novel Object Recognition Test

Two identically shaped objects were placed in a 60 cm × 60 cm cubic chamber. A mouse was placed in the chamber and allowed to freely explore for 10 min. Six hours later, one of the objects was substituted with a novel object. The mouse was then placed in the chamber for another 10 min, and the mouse behavior was recorded throughout the experiment. The accumulated time that each mouse spent around each object was measured by EthoVision XT 14.

#### 2.4.8. Morris Water Maze Test

A Morris water maze test was conducted in a circular pool (90 cm diameter with 40 cm depth) filled with water at a temperature of 25 °C, and skim milk was added to make the water translucent. The pool was equally divided into four sections (I, II, III, IV). A platform was placed in the middle of target quadrant I (12 cm from the pool’s edge and 0.5 cm below the surface of the water) with visual cues on the wall as spatial references. Mice received an acquisition phase training protocol for 9 consecutive days: cue training (3 days) followed by spatial training (6 days). Four trials were conducted on each mouse per day, and the escape latency (time to reach the platform) in each trial was measured. For each trial, the subject mouse was gently placed into the water facing toward the wall from one of three quadrants, varied by days of trial. Each mouse was given 1 min to find the platform. If any mouse failed to find the platform within 1 min, the mouse was guided to the platform by the investigator and a 30 s resting time was given. The mean of the escape latencies for four trials was represented for the learning result for each mouse. Recording took place on the 10th day, the platform was removed from the experimental pool, and the mice were left to freely explore the pool for 1 min. Videos were recorded and analyzed using EthoVision XT 14. The time spent in the target platform area and the number of times the platform area was crossed were measured to assess the memory ability.

### 2.5. Data Analysis

Data were analyzed with an ordinary one-way analysis of variance (ANOVA), using Dunnett’s multiple comparisons test to compare each group to the vehicle group. All statistical analyses were performed using GraphPad Prism 8 for Windows. *p* < 0.05 was considered statistically significant.

## 3. Results

This section is divided into subsections. It should provide a concise and precise description of the experimental results, their interpretation, and the experimental conclusions that can be drawn.

### 3.1. D5 Is a Developmental Neurotoxicant

**Figure 2:**
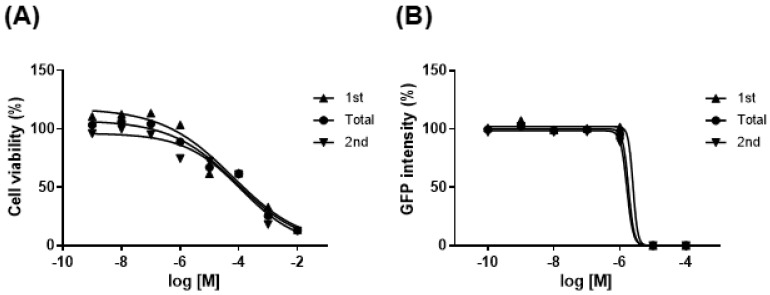
Discrimination of D5 as a developmental neurotoxicant by DNT: (**A**) cell survival curve of 46C; (**B**) logarithmic curve of the fluorescence intensity of GFP in neurosphere.

The cell viability (IC_50_) of Sox1-GFP cells (46C) was evaluated using a CCK assay (Figure 2A). The ID_50_ of D5 was confirmed (Figure 2B); the IC_50_ is the half-maximal inhibitory concentration of the biological function by a chemical, and the ID_50_ is half the inhibitory concentration for the neural differentiation of 46C. The obtained values were placed into the discriminant function previously established by Park et al. [19]. The discriminant function score was −1.55603. The negative value of the discrimination function score implies that D5 is a developmental neurotoxicant.

### 3.2. Maternal D5 Exposure Induced Abnormal Behavior in Offspring Mice

#### 3.2.1. D5 Induced Repetitive Activity

**Figure 3:**
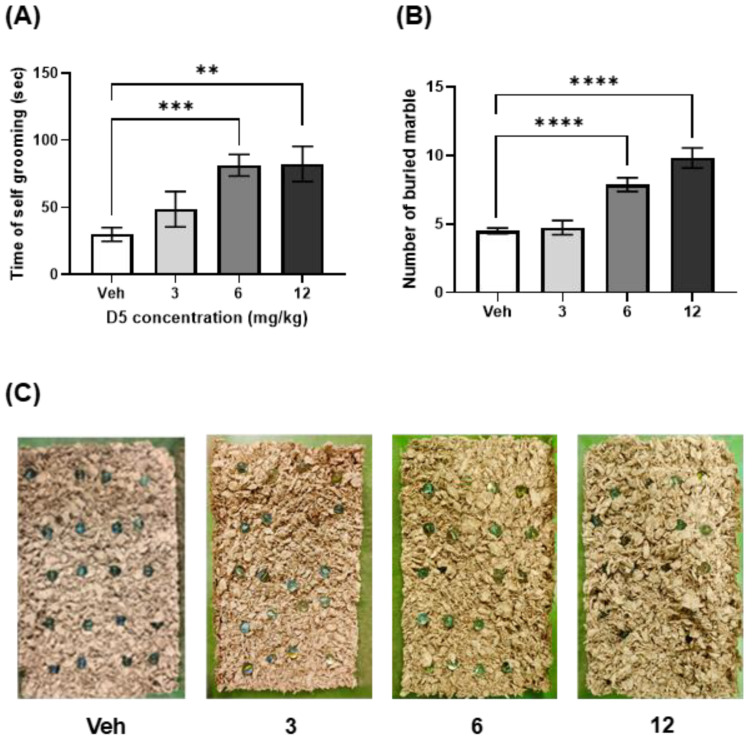
Effect of D5 exposure on repetitive activity in offspring mice: (**A**) bar graph of self-grooming time in the grooming test; (**B**) bar graph of buried marble number in the marble-burying test. (**C**) Representative pictures of the marble-burying test. Significance was obtained using an ANOVA test. ** *p* < 0.01, *** *p* < 0.001 and **** *p* < 0.0001 vs. vehicle. Each value is expressed as the means ± SEM. Vehicle (*n* = 20), D5 3 mg/kg/day (*n* = 14), D5 6 mg/kg/day (*n* = 33), and D5 12 mg/kg/day (*n* = 30). Veh, vehicle.

The grooming test was conducted to assess compulsive, repetitive activity. The accumulated self-grooming time representing the repetitive behavior was significantly higher in the high-dose group than in the vehicle group (Figure 3A). The standard dose group also showed a significantly higher accumulated self-grooming time than the vehicle group (Figure 3A). The marble-burying test result measures the compulsive, repetitive activity. The number of buried marbles was significantly increased in the high-dose group than in the vehicle group (Figure 3B,C).

These results show that maternal D5 exposure induced repetitive and compulsive activity in offspring mice.

#### 3.2.2. D5 Induced Depression-like Behavior

**Figure 4:**
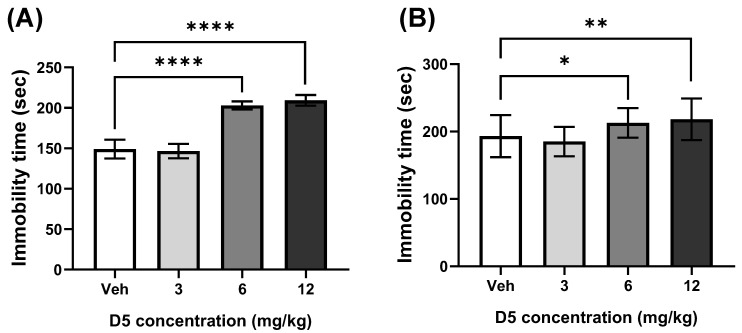
Effect of D5 exposure on depression in offspring mice: (**A**) bar graph of immobility time in the tail suspension test; (**B**) bar graph of the immobility time in the forced swimming test. The significance was obtained using an ANOVA test. * *p* < 0.05, ** *p* < 0.01 and **** *p* < 0.0001 vs. vehicle. Each value is expressed as the means ± SEM. Vehicle (*n* = 20), D5 3 mg/kg/day (*n* = 14), D5 6 mg/kg/day (*n* = 33), and D5 12 mg/kg/day (*n* = 30). Veh, vehicle.

The tail suspension test and forced swimming test were conducted to determine whether maternal D5 exposure causes depression-like behavior. The accumulated immobility time represents the depression level. The 12 mg/kg group showed significantly increased depression-like behavior compared to the vehicle group (Figure 4A). The 6 mg/kg group also had a longer immobility time compared to the vehicle group (Figure 4A). There was no difference between the 3 mg/kg group and the vehicle group (Figure 4A).

The forced swimming test was conducted to confirm the effects of maternal D5 exposure on the depression level. The accumulated immobility time was regarded as the level of depression-like behavior. The results show that the immobility time was significantly higher in the 12 mg/kg group than in the vehicle group (Figure 4B). The 6 mg/kg group showed significantly higher depression-like behavior (Figure 4B). The result of the 3 mg/kg group was similar to the vehicle group (Figure 4B). Overall, maternal D5 exposure increased the depression level in the offspring mice. These results show that maternal D5 exposure induced depression-like behavior in offspring mice.

#### 3.2.3. D5 Impaired Sociality in Offspring Mice

**Figure 5:**
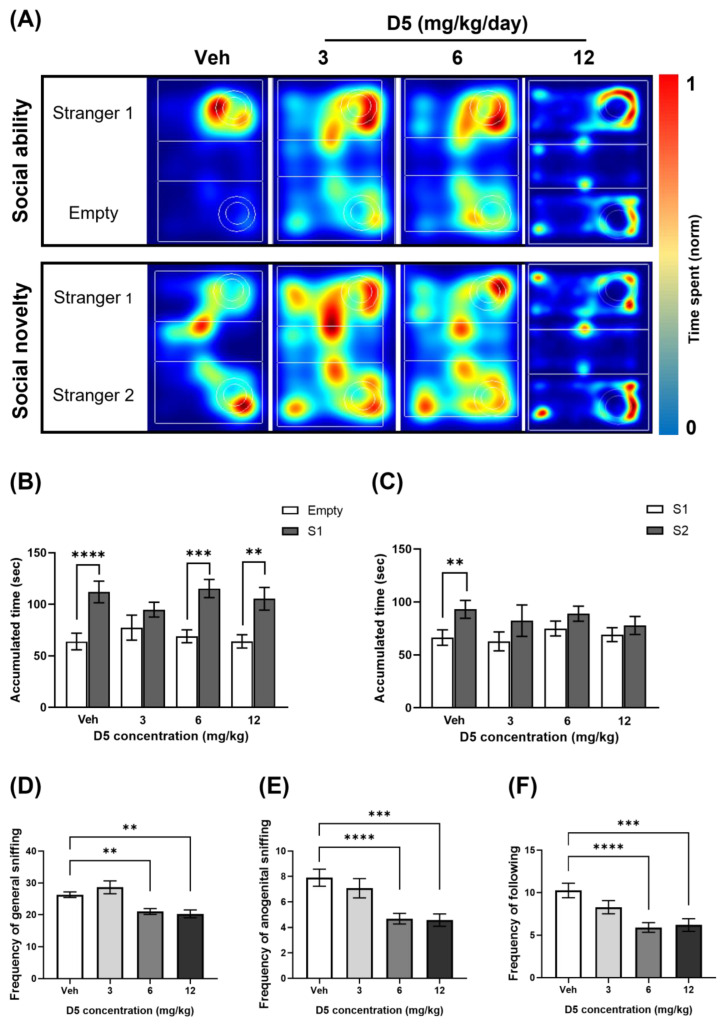
Effect of D5 exposure on sociality in offspring mice: (**A**) image of a heatmap of the accumulated time at the location around the cylinder-shaped cage in the three-chamber test; (**B**) bar graph of the accumulated time (AT) at the location around the cylinder-shaped cage in the social ability test of the three-chamber test and (**C**) in the social novelty test; bar graphs of the frequency of (**D**) general sniffing and (**E**) anogenital sniffing in the social interaction test; (**F**) bar graph of the frequency of following the social interaction test. Significance was obtained using an ANOVA test. ** *p* < 0.01, *** *p* < 0.001 and **** *p* < 0.0001 vs. vehicle. Each value is expressed as the means ± SEM. Vehicle (*n* = 20), D5 3 mg/kg/day (*n* = 14), D5 6 mg/kg/day (*n* = 33), and D5 12 mg/kg/day (*n* = 30). Veh, vehicle.

In the three-chamber test, the social ability test and social novelty test were conducted to assess the sociability and memory. In the social ability test, the mice were permitted to explore the three chambers in the presence of a stranger mouse in one of the side chambers (Figure 5A). The vehicle group spent significantly more time around the cage containing stranger mouse 1 (S1) than the empty cage (E) (Figure 5A,B). Furthermore, the 6 mg/kg and 12 mg/kg groups spent more time around the S1 cage. However, there was no significant difference in the time that mice spent around the cage between the S1 cage and the empty cage in the 3 mg/kg group (Figure 5C).

In the social novelty test, stranger mouse 2 (S2) was introduced into the empty cage (Figure 5A). In this case, the vehicle group showed a greater preference for S2 than the familiar S1 (Figure 5A,C). However, there were no significant differences in social novelty in all D5-treated groups (Figure 5C).

In the social interaction test, the 6 mg/kg group and 12 mg/kg group showed lower social behaviors than the vehicle group (Figure 5D–F). The 6 mg/kg group and 12 mg/kg group showed lower frequencies of general sniffing, anogenital sniffing, and following than the vehicle group. There were no significant differences in the frequency of general sniffing, anogenital sniffing, and following in the 3 mg/kg group compared with the vehicle group (Figure 5D–F).

The results show that maternal D5 exposure impaired the sociality of offspring mice.

#### 3.2.4. D5 Impaired Memory and Learning Ability

**Figure 6:**
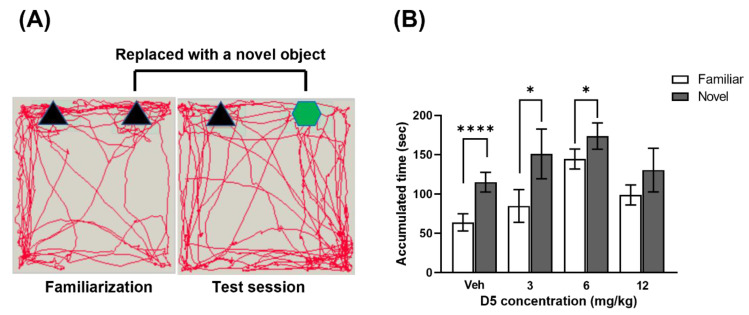
Effect of D5 exposure on memory ability in offspring mice. (**A**) Method of the novel object recognition test. (**B**) Bar graph of the accumulated time ratio of mice remaining in the area around the object. Significance was obtained using an ANOVA test. * *p* < 0.05 and *p* **** < 0.0001 vs. vehicle. Each value is expressed by the mean ± SEM. Vehicle.

A novel object recognition test and Morris water maze test were conducted to assess the memory and learning ability. In the novel object test, the accumulated time spent in the vicinity of each object was evaluated. The vehicle group took much more interest in the novel object than the familiar object (Figure 6B). The 3 mg/kg group and 6 mg/kg group also showed more interest in the novel object than in the familiar object (Figure 6B). By contrast, the 12 mg/kg group showed no difference in interest between the novel object and familiar object (Figure 6B). Hence, the result shows that the 12 mg/kg group could not differentiate between the novel object and the familiar object.

**Figure 7:**
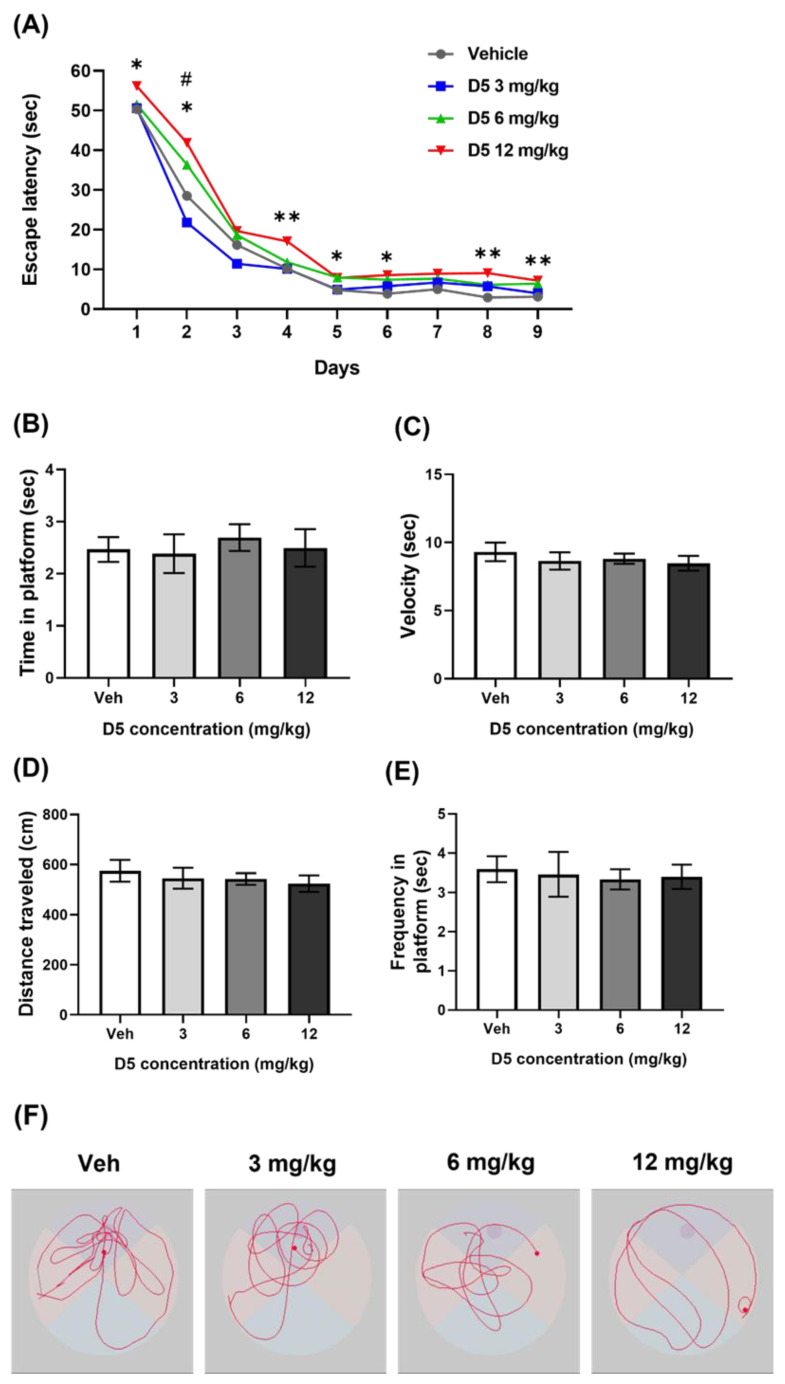
Effect of D5 exposure on learning ability in offspring mice: (**A**) Time escaping to the latency platform; (**B**) bar graph of the time to reach the location where the platform was; (**C**) bar graph of the velocity of mouse traveling to the experimental pool; (**D**) bar graph of distance traveled; (**E**) bar graph of the frequency of mouse entering the location where the platform existed; (**F**) image of the mouse movement tracking. Significance was obtained using ANOVA test. * *p* < 0.05, ** *p* < 0.01, 12 mg/kg vs. vehicle. # *p* < 0.05, 6 mg/kg vs. vehicle. Each value is expressed as the means ± SEM. Vehicle (*n* = 20), D5 3 mg/kg/day (*n* = 14), D5 6 mg/kg/day (*n* = 33), and D5 12 mg/kg/day (*n* = 30). Veh, vehicle.

Next, the Morris water maze test was conducted to evaluate the memory and spatial learning ability. Compared to the vehicle group, the 12 mg/kg group took more time to find the platform during the final acquisition phase (Figure 7A). The 6 mg/kg group took more time to find the platform until the acquisition phase on day 2 (Figure 7A). On days 3–9 of the acquisition phase, the 6 mg/kg group performed similarly to the vehicle group (Figure 7A). On the other hand, the 12 mg/kg group spent significantly more time in the acquisition phase days 1–2, 4–6, and 8–9 than the vehicle group. The time spent finding the platform was similar in the 3 mg/kg and vehicle groups throughout the entire acquisition phase (Figure 7A). This result indicates that the spatial learning capacity in the 6 mg/kg and 12 mg/kg groups was delayed.

After the acquisition phase, the learning ability was evaluated. The platform was removed from the experimental pool, and the speed, route, distance traveled, and duration of staying in the place where the platform used to exist were measured. However, there were no significant differences in performance throughout all groups regarding the velocity, distance moved, time of staying in place, and frequency of crossing the location where the platform existed (Figure 7B). The visual representation recorded for all swim tracks of each group showed that the D5-treated groups had lower proximities to the area where the platform was originally located than the vehicle group (Figure 7C). Overall, maternal D5 exposure impaired the spatial learning speed.

## 4. Discussion

This is the first study, to the best of our knowledge, demonstrating that maternal D5 exposure induces behavioral abnormalities in offspring. D5 is a key ingredient in many toiletries and cosmetics and is a precursor in the manufacture of siloxane polymers for industry and medicine. A few studies have shown that D5 has no estrogenic or androgenic effect [20,21] and even reproductive toxicity in a two-generation study [22]. On the other hand, some studies have reported changes in the amounts of prolactin, progesterone, estradiol, and corticosterone, along with dopamine [17]. In the inhalation test with rats, chronic exposure to D5 with NOAEL-dose nasal epithelium cells changed the immune response of the respiratory tract [20]. Moreover, the liver weight was increased in the inhalation test [1,23]. However, with chronic exposure, the liver weight recovered by 24 months later. Even though the liver weight was recovered in the chronic test, alterations in enzymes and liver damage were observed [1,18]. These results suggest that D5 affects the body via an unknown pathway. Although it has a slight effect on a mature body, it can profoundly affect the differentiation or proliferation of immature cells, as found in our study, which might affect embryonic stem cells.

In this experiment, we examined whether maternal D5 exposure causes neurodevelopmental disorders through behavioral experiments. One of the representative neurodevelopmental disorders, autism spectrum disorder is known to cause repetitive behaviors and limited social skills [24]. ADHD is also one of the diseases caused by neurodevelopmental disorders, and it is known to cause defects in learning abilities [25]. In this study, the degree of neurodevelopment was evaluated by assessing the behavioral phenomena of ASD and ADHD. As a result, offspring mice exposed to maternal D5 showed abnormalities compared to vehicle mice in repetitive activity, depression-like behavior, memory, and learning ability. In particular, in the three-chamber test, used to examine the social ability, mice were not able to distinguish a familiar mouse from a stranger mouse in all groups except for the vehicle group, and it is presumed that the maternal D5 exposure had a great influence on the neural circuit responsible for social behavior. There is a report that these behavioral changes are caused by a decrease in dopamine, which leads to a decrease in learning ability and a decrease in sociality [26,27].

Octamethylcyclotetrasiloxane (D4), which has a similar structure to D5, is also assumed to be released into the environment equivalently. D4 is known as an EDC and showed similar adverse effects as D5 [3]. In a previous study, prenatal D4 exposure induced abnormal behaviors such as decreased motor function, cognition, and social ability in behavioral tests. These similarities may be caused by their structural similarity. Hence, we suggest that D5 has a similar effect to D4. In the study, D4 caused the impairment of neural progenitor cell proliferation. According to those results, decreased neural progenitor cell viability might be the cause of the proliferation impairment effect of D5. In the past, the toxicity of D5 was evaluated with the NOAEL dose [23,28,29]. However, EDCs such as D4 and bisphenol A have non-monotonic dose–response relationships [30]. Since D5 has a similar structure to D4, the possibility of D5 showing a non-monotonic dose–response relationship cannot be ruled out. To verify this possibility, the minimum range of drug treatment dosage was evaluated. Hence, D5 was administrated at 3, 6, or 12 mg/kg daily to mice. This concentration is considered a reasonable dosage because, according to a report evaluating D5 exposure, the estimated daily exposure amount of D5 was 0.6 mg/kg [2].

The developmental neurotoxicity of D5 was evaluated using the DNT method [19]. This method supported the results from in vivo tests that D5 has neurotoxicity. In addition, D4 was reported to be a neurotoxicant and induce behavioral abnormalities in offspring exposed at the maternal stage [16]. Recently, the association of EDCs and neurodevelopmental disorder has been unveiled, and EDCs have been suggested as one of the factors causing autistic behavior [28,31]. The results reported by Tran for D4 [16] and the present result for D5 support this hypothesis.

Furthermore, some chemicals known as developmental neurotoxicants induce abnormal behaviors in offspring. Bisphenol A(BPA), known as a developmental neurotoxicant and also an EDC, induces alterations in murine behaviors such as increasing locomotive activity and impaired memory [29]. Moreover, prenatal BPA exposure causes cognitive deficits and anxiety-like behavior in mice [32]. Additionally, well-known neurotoxicants such as chlorpyrifos and lead have been shown to induce abnormal behaviors resembling typical symptoms of neurodevelopmental disorder including attention deficit–hyperactivity disorder (ADHD), autism spectrum disorder (ASD), and obsessive-compulsive disorder (OCD) [33,34,35,36]. The alterations in offspring behavior in these studies as well as in our research suggest that D5 might cause developmental disorders. Additionally, as the lower-dose treatment of D5 seems more efficient than the higher dose, the effect of D5 as an EDC should be re-evaluated with a much lower dose than the NOAEL dose. Future research should investigate the mechanism by which D5 impairs neural development.

In conclusion, D5 has been used without strict limitations in real life because its safety has been unknown and its chemical composition is relatively safe. This study suggests that even the dose that humans are routinely exposed to could cause abnormal behavior in offspring. Thus, the use of D5 needs to be considered carefully, and warnings should be given. It is also necessary to carry out additional research into the neurodevelopmental toxicity of D5 and the mechanism by which D5 induces neurodevelopmental disorder on a molecular level.

## Data Availability

Publicly available datasets were analyzed in this study. This data can be found here: [https://www.cyclosiloxanes.org/uploads/Modules/Links/10.-environment-and-health-canada-screening-assessment-for-the-challenge-decamethylcyclopentasiloxane-(d5).pdf/541-02-6 (accessed on 14 November 2022)]. Publicly available datasets were analyzed in this study. This data can be found here: [https://www.canada.ca/en/environment-climate-change/services/archive/octamethylcyclotetrasiloxane.html/556-67-2 (accessed on 14 November 2022)].
